# Segmentation and morphological analysis of recanalized coiled aneurysms: Towards an automatic tool for clipping planning in microsurgery

**DOI:** 10.1371/journal.pone.0358945

**Published:** 2026-09-23

**Authors:** Matthieu Coupet, Manel Meftah, Camille Lopez, Ludivine Maintier, Aziliz Guezou-Philippe, Julien Ognard, Jean-Christophe Gentric, Valérin Burdin, Elsa Magro, Guillaume Dardenne

**Affiliations:** 1 LaTIM - UMR 1101 INSERM, Brest, France; 2 IMT Atlantique, Brest, France; 3 Department of Neurosurgery, Hôpital Cavale Blanche, Brest University Hospital, Brest, France; 4 Department of Radiology, Hôpital Cavale Blanche, Brest University Hospital, Brest, France; 5 Service d’Imagerie Médicale, Hôpital d’Instruction des Armées Legouest, rue des Frères-Lacretelle, Metz, France; 6 University of Brest GETBO - INSERM UMR1304, Brest, France; Università degli Studi di Napoli Federico II: Universita degli Studi di Napoli Federico II, ITALY

## Abstract

Intracranial aneurysms are dilatations of cerebral arteries that may rupture, leading to subarachnoid hemorrhage and high mortality. Endovascular coiling is widely used to prevent rupture; however, many treated aneurysms experience recanalization, which often necessitates follow-up interventions or surgical clipping. Despite its clinical importance, the segmentation and morphological characterization of recanalized coiled aneurysms remain largely unexplored. In this study, we introduce an automated framework for the segmentation of recanalized coiled aneurysms from three-dimensional rotational angiography (3D-RA) images and the extraction of morphological descriptors relevant to surgical planning. The method is based on the nnU-Net architecture, trained to segment the vascular tree, coil mass, and recanalized lumen. Quantitative postprocessing enables the computation of clinically relevant parameters, including coil–neck distance, recanalization volume, and maximum neck diameter, which contribute to the assessment of clipping feasibility. Preliminary results show encouraging segmentation performance and consistent estimation of morphological parameters across the evaluated cases, supporting the potential of the proposed framework for preoperative assessment of recanalized coiled aneurysms.

## Introduction

Intracranial aneurysms (IA), also known as cerebral aneurysms, are a deformation of the cerebral artery wall, leading to a fragile arterial zone that can rupture. Aneurysmal rupture systematically causes a subarachnoid hemorrhage (SAH) of variable severity, which may result in death or severe neurological sequelae. It is estimated that one-third of patients with this condition will die, one-third will survive with a poor neurologic outcome, and one-third will have a favorable outcome. Approximately 3.2% of the global population has a cerebral aneurysm, with rupture being fatal in approximately 30% of cases [1,2]. The historical treatment of IAs is surgical clipping via an extravascular approach requiring craniotomy. Although the technique has evolved with the refinement of micro-instrumentation and the use of an operating microscope, less invasive treatments using coils guided by endovascular imaging in interventional neuroradiology have become increasingly predominant [3].

Coiled intracranial aneurysms carry a significant risk of recanalization, reported in up to 20.8% of cases over a follow-up period of 4.7 to 38 months [4].

In cases of recanalization, microsurgical treatment is effective and safe [5] but the treatment of previously embolized aneurysms presents additional challenges. The presence of metallic coils within the aneurysmal sac creates mechanical restrictions and limits wall deformation at the aneurysm neck, making clipping impossible in some cases without risk to the parent artery or its branches. In some cases, it may even be necessary to wait for increased recanalization before performing microsurgery, which exposes the patient to the risk of aneurysmal rupture and its potentially serious clinical consequences.

Currently, 3D reconstruction of a recanalized aneurysm is possible using data from 3D Rotational Angiography (3D-RA) and serves as a tool to support surgical feasibility decision-making. However, this reconstruction has limitations due to variability in the morphology of the aneurysmal sac and its branching vessels, as well as variability in wall thickness, rigidity, and position of endovascular material within the sac. Morphological measurements taken from the 3D reconstructions guide therapeutic decision-making. These measurements are performed manually and are subject to significant variability between operators [6]. Therefore, there is a strong interest in developing reliable automated segmentation and morphometric analysis for both untreated and recanalized aneurysms. More broadly, computational tools have also been investigated to support cerebral aneurysm treatment planning. For example, Briganti et al. [7] explored virtual stenting as a simulation-based approach to assist endovascular treatment planning and clinical decision-making.

Regarding automated segmentation, artificial intelligence (AI) techniques are increasingly used for this purpose [8,9] and, in particular, deep learning-based approaches such as U-Net-based methods which have shown strong performance [10,11]. More recently, various challenges such as the 2018 Multiple AneurysmsAnaTomyCHallenge (MATCH) [12], the 2020 Aneurysm Detection and SegMentation (ADAM) [13], or the 2020 Aneurysm Detection and Analysis (CADA) [14] challenges have been organized to provide data and compare the performance of the methods for automatic detection and segmentation of intracranial aneurysms. However, despite the latest improvements supported by these challenges, none of these approaches have been developed for coiled aneurysms.

Regarding automated morphometric analysis, Nishi et al. proposed a solution to compute five morphological parameters from angiography, that is, the aneurysm volume, maximum aneurysm diameter, neck diameter, aneurysm height, and the aspect ratio of aneurysm height by neck size [15]. Jerman et al. developed an automatic cut-plane positioning method for neck delineation [16]. Saalfeld et al. developed a semi-automatic 3D method to reconstruct the neck curve of aneurysms that allows automatic extraction of 20 morphological parameters, including the delineation of the neck plane [17]. However, none of these approaches have been tested with coiled aneurysms.

A targeted PubMed search using combinations of terms related to intracranial aneurysms, coiling, recanalization, segmentation, morphometry, automation, 3D rotational angiography, clipping, and surgical planning identified no equivalent framework specifically addressing automated segmentation and morphometric analysis of recanalized coiled aneurysms for microsurgical clipping planning. The main contributions of this work are: (i) an automated nnU-Net-based segmentation of the vascular tree, coil mass, and recanalized lumen from 3D-RA images; and (ii) a morphometric analysis of the recanalized aneurysm, including neck characterization, coil-to-neck distance, and recanalization volume, designed to support preoperative assessment for microsurgical clipping.

## Materials and methods

**Fig 1:**
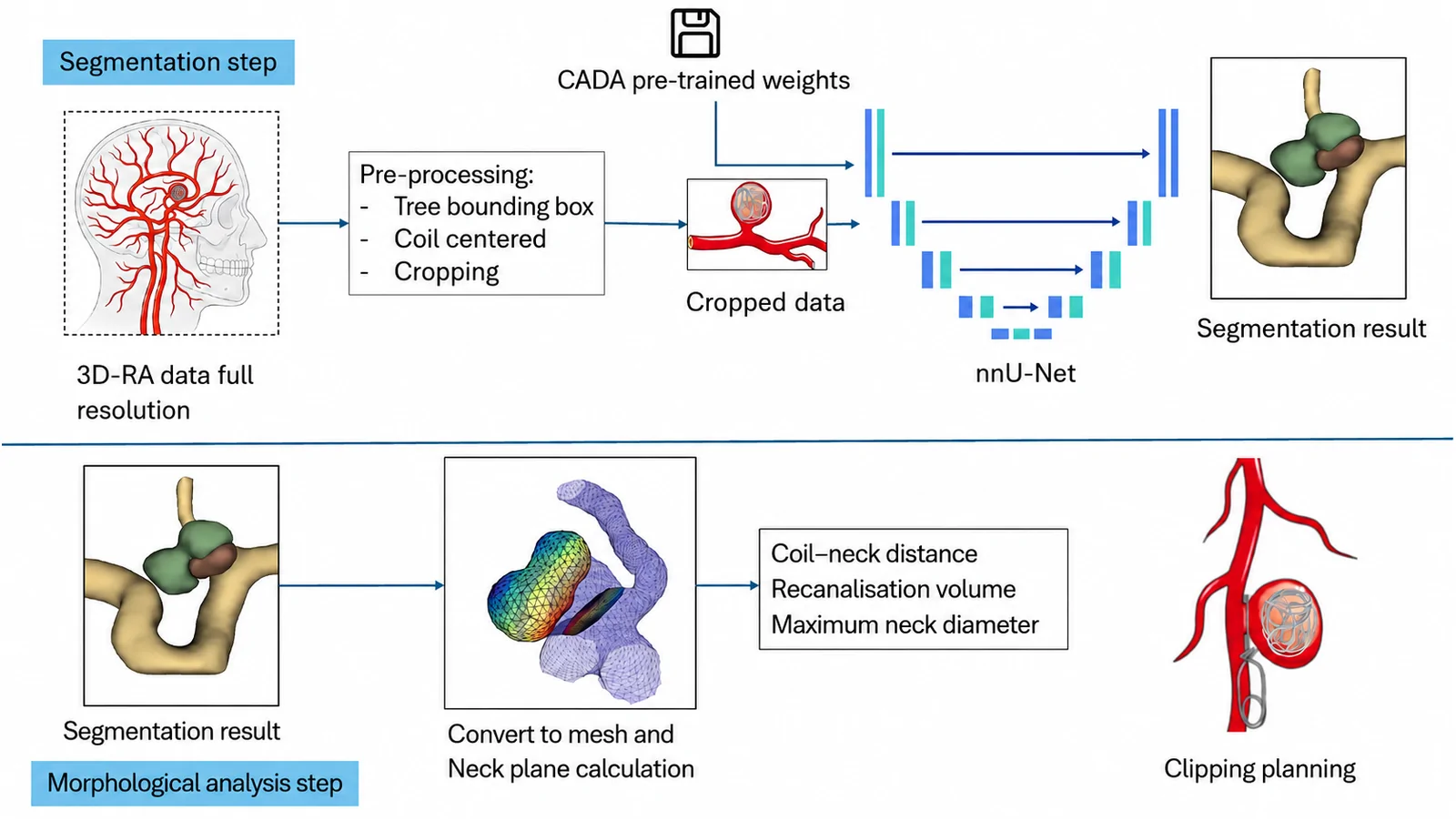
Workflow for the automatic segmentation and morphological analysis of recanalized coiled aneurysms for microsurgical clipping planning. The upper panel illustrates the segmentation pipeline, from 3D-RA data pre-processing and cropping to nnU-Net-based segmentation. The lower panel shows the morphological analysis step, including mesh conversion, neck plane calculation, and extraction of morphometric parameters (coil–neck distance, recanalization volume, and neck maximum diameter), which support clipping strategy planning.

An overview of the global workflow is presented in Figure 1. There are two main tasks: the automatic segmentation of coiled aneurysms and their morphometric analysis. The proposed segmentation approach allows for the accurate delineation of the key anatomical structures of interest, including the parent artery, the aneurysm sac containing coil material and, most importantly, the recanalized compartment. This segmentation enables the extraction of precise morphological features, which are essential to evaluate whether surgical clipping remains anatomically feasible, such as the coil neck distance, the recanalization volume, and the maximum diameter of the neck.

### Database

The study protocol (PREDICTNRI, code 29BRC20.0032) received a favorable opinion from the Ethics Committee of Brest (reference B2020CE.06) on January 30, 2020. Patient inclusion for the cohort considered in this study took place between 01/05/2015 and 31/12/2023 using a retrospective protocol. All participants (or their legal representatives) provided written informed consent prior to inclusion, except in emergency situations where consent was obtained as soon as possible. Within PREDICTNRI, which was registered on ClinicalTrials.gov (NCT04504695), 3D-RA images of treated patients with recanalization and complete available examinations were collected from the Picture Archiving and Communication System (PACS) of Brest University Hospital and anonymized, in DICOM format. Patients under 18 years of age, with nonsaccular aneurysms, or treated with devices other than coils were excluded from the study, as well as patients with aneurysms not representative of typical recanalization. 3D-RA were acquired using a biplane scanner (Siemens Artis Q, Siemens Healthcare, Erlangen, Germany) with automatic injection of contrast product (IOMERON 250, Bracco Imaging, Milan, Italy) with an average of 383 images of 512×512 pixels per patient and a voxel spacing of 0.460 mm.

To our knowledge, no publicly available dataset provides 3D-RA images of recanalized coiled aneurysms together with expert three-dimensional annotations of the vascular tree, coil mass, and recanalized compartment. The dataset therefore had to be assembled retrospectively from this highly specific clinical population, substantially limiting the number of eligible cases.

All manual reference annotations were performed by a single qualified neurosurgeon from the University Hospital of Brest using ITK-SNAP (version 3.8.0). For each case, three classes were annotated: the vascular tree, the coil, and the recanalized lumen. The vascular annotation was task-oriented, prioritizing the parent vascular structures relevant to aneurysm assessment and clipping planning; small collateral vessels or vessels crossing the region of interest were therefore not systematically annotated.

The final data set was divided into two subsets: a training set comprising 63 scans (33 coiled + 30 non-coiled), and a test set containing 16 scans, all of which were coiled aneurysms. Segmentation performance was quantitatively evaluated on an independent test set of 16 coiled aneurysm cases that were not used for model training or validation. Two additional cases, subsequently annotated by the neurosurgeon, were included only in the downstream morphometric evaluation, resulting in a total of 18 cases for this analysis.

### Segmentation

The segmentation process is divided into two steps: pre-processing and classification.

Given the large size of the images, a region of interest (ROI) was first computed around the coiled aneurysm. The cranial cavity was localized by identifying the largest connected component with Hounsfield Unit (HU) values between 1000 and 2000, corresponding predominantly to cranial bone, and a square bounding box centered on the skull was then defined. This first spatial restriction was used to exclude extracranial highly radiopaque structures, particularly metallic dental fillings, which could otherwise be mistaken for coil material. Within this cranial bounding box, the high and relatively homogeneous attenuation of the coils was exploited by applying a threshold of 9500 HU. The resulting coil candidates were then used to compute a second bounding box centered on the coiled aneurysm.

**Fig 2:**
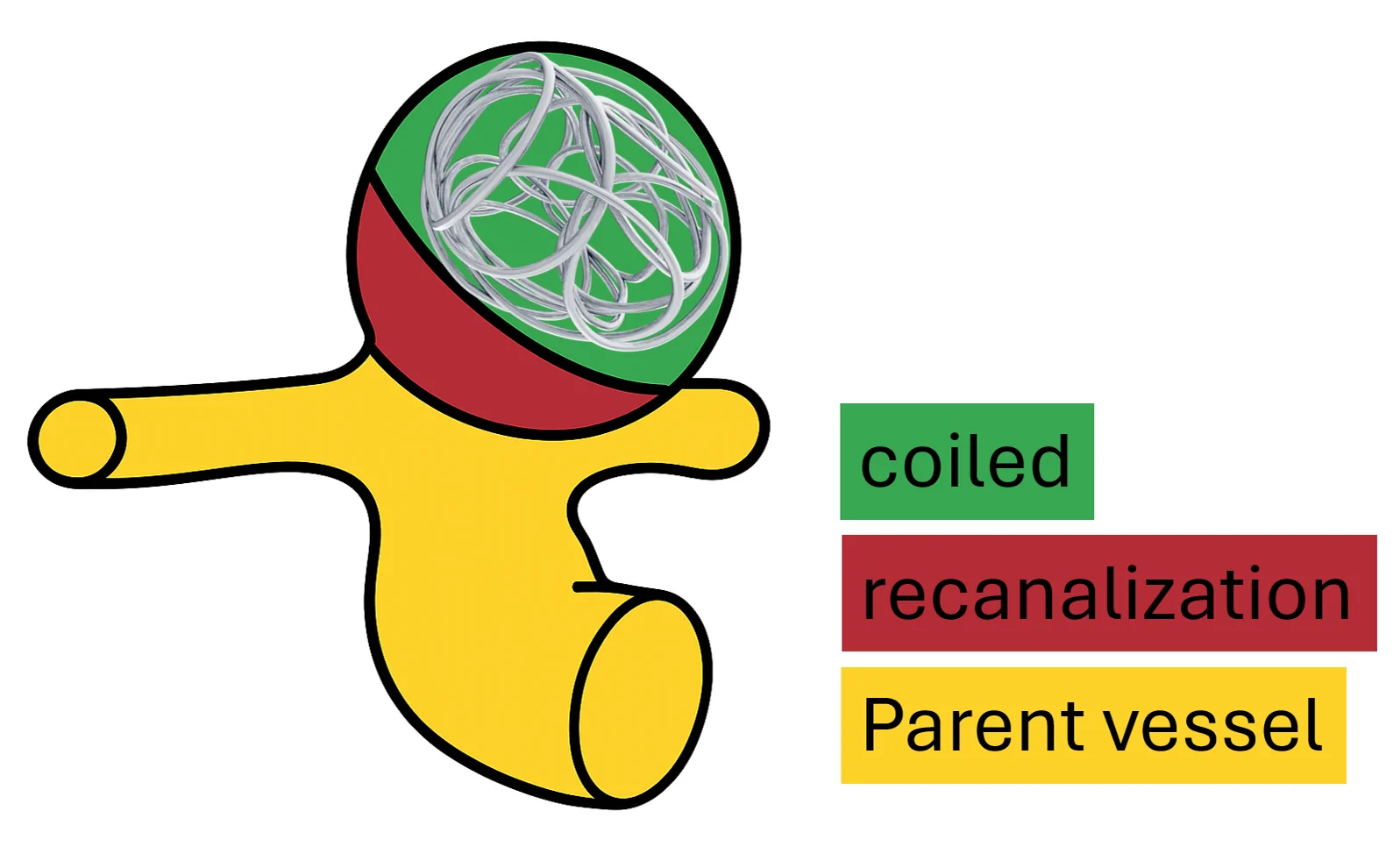
Schematic representation of the relevant anatomical structures: the parent artery (yellow), the coiled aneurysm sac (green), and the recanalization volume (red).

From these bounding boxes, the nnU-Net framework was then used to segment the coil, the recanalization area, and the vascular tree. Fig 2 illustrates the three main components to label: the parent vessel (yellow), the coiled aneurysm sac (green), and the recanalization volume (red). The full-resolution 3D U-Net configuration that operates on the entire volume using 3D convolutional layers was used for this purpose.

The pretraining was conducted first on CADA data, including uncoiled aneurysms, using the fold that showed the best performance in the dataset provided by [18]. This step aimed to help the network learn both the vascular architecture and the morphology of the aneurysm. Subsequently, training was performed on our development cohort comprising both coiled and non-coiled aneurysms. A five-fold cross-validation strategy was used, with one-fifth of the data used for validation at each iteration. Training lasted 500 epochs, starting with a learning rate of 0.01, and used a combined Dice loss and cross-entropy loss.

To assess segmentation performance, two commonly used overlap metrics were selected: the Dice similarity coefficient, which measures spatial overlap between two segmentations, and the Jaccard coefficient, which measures their intersection over union [19].

### Morphometric analysis

Based on the segmentation, morphometric analysis was performed by assessing three key clinical parameters relevant to the feasibility of clip placement: (1) the distance between the coils and the aneurysm neck, (2) the recanalization volume, and (3) the maximum neck diameter.

Two of these parameters, the coil–neck distance and maximum neck diameter, require prior estimation of the aneurysm neck. The approach described by Jerman et al. [16] was used and is based on several steps: the estimation of both the centerline of the parent artery and the center of the aneurysm sac, thereby allowing determination of the aneurysm’s principal orientation, which is then used to estimate the neck by minimizing a cost function.

The distance between the coils and the aneurysm neck is measured by computing a distance map between the neck surface and the coil surface to obtain the coil–neck distance at each point of the neck surface. Fig 5 shows a visual analysis of this approach.

The recanalization volume is obtained by counting voxels and converting the measurements according to voxel spacing.

The maximum diameter of the neck was calculated from the convex hull of the points after alignment of the neck plane, by determining the maximum distance between any two points on this convex hull.

The coil volume was additionally calculated as a complementary descriptor to quantify the amount of residual endovascular material relative to the newly perfused compartment. This parameter provides insight into the spatial relationship between the coil mass and the recanalized lumen, which is critical for planning surgical clipping. A large coil volume can also alter the mechanical properties of the aneurysm sac, making it stiffer and less malleable, thereby complicating clip placement and influencing the choice of surgical strategy.

Together, the three primary parameters—coil–neck distance, recanalization volume, and maximum neck diameter—provide the complete morphometric information required to assess clipping feasibility within the proposed planning framework. Subsequent developments may use this information to assist with clip selection and positioning.

To validate the proposed algorithm, the differences between the manual reference measurements and the automatic estimates (including the distance map, recanalization volume, and maximum neck diameter) were calculated and analyzed, providing an evaluation of the accuracy of the proposed morphometric approach. The morphometric evaluation was conducted on 18 cases, comprising the 16-case independent segmentation test set and two additional cases subsequently annotated for downstream analysis. Manual measurements performed by an experienced clinician using 3D Slicer served as the reference standard for comparison.

## Results

### Segmentation

Table 1 summarizes the performance metrics of the 3D nnU-Net frameworks pre-trained on CADA weights, specifically focusing on the Dice score and Jaccard index for three distinct anatomical structures: coils, vascular trees, and recanalization areas. The mean results were derived from a set of 16 test cases.

**Table 1:** Segmentation performance on the independent test set (*n* = 16). Values are reported as mean with 95% confidence intervals (CI).

Structure	Dice, mean (95% CI)	Jaccard, mean (95% CI)
Coil	0.878 (0.783–0.943)	0.788 (0.769–0.876)
Vascular tree	0.876 (0.779–0.923)	0.790 (0.774–0.869)
Recanalization area	0.783 (0.693–0.822)	0.659 (0.633–0.801)

The Dice score indicates high performance across all categories, with the coil achieving a score of 0.878 and the vascular tree closely following at 0.876. The recanalization area, while still demonstrating reasonable performance, recorded a lower Dice score of 0.783.

The vascular tree obtained the highest Jaccard index of 0.790, suggesting effective delineation, while the coil and recanalization area recorded indices of 0.788 and 0.659, respectively. A lower Jaccard index was observed for the recanalization area.

On the independent test set of 16 cases, mean Dice scores were 0.878, 0.876, and 0.783 for the coil, vascular tree, and recanalization area, respectively. Corresponding mean Jaccard indices were 0.788, 0.790, and 0.659. The associated 95% confidence intervals are reported in Table 1.

### Morphometric analysis

**Fig 3:**
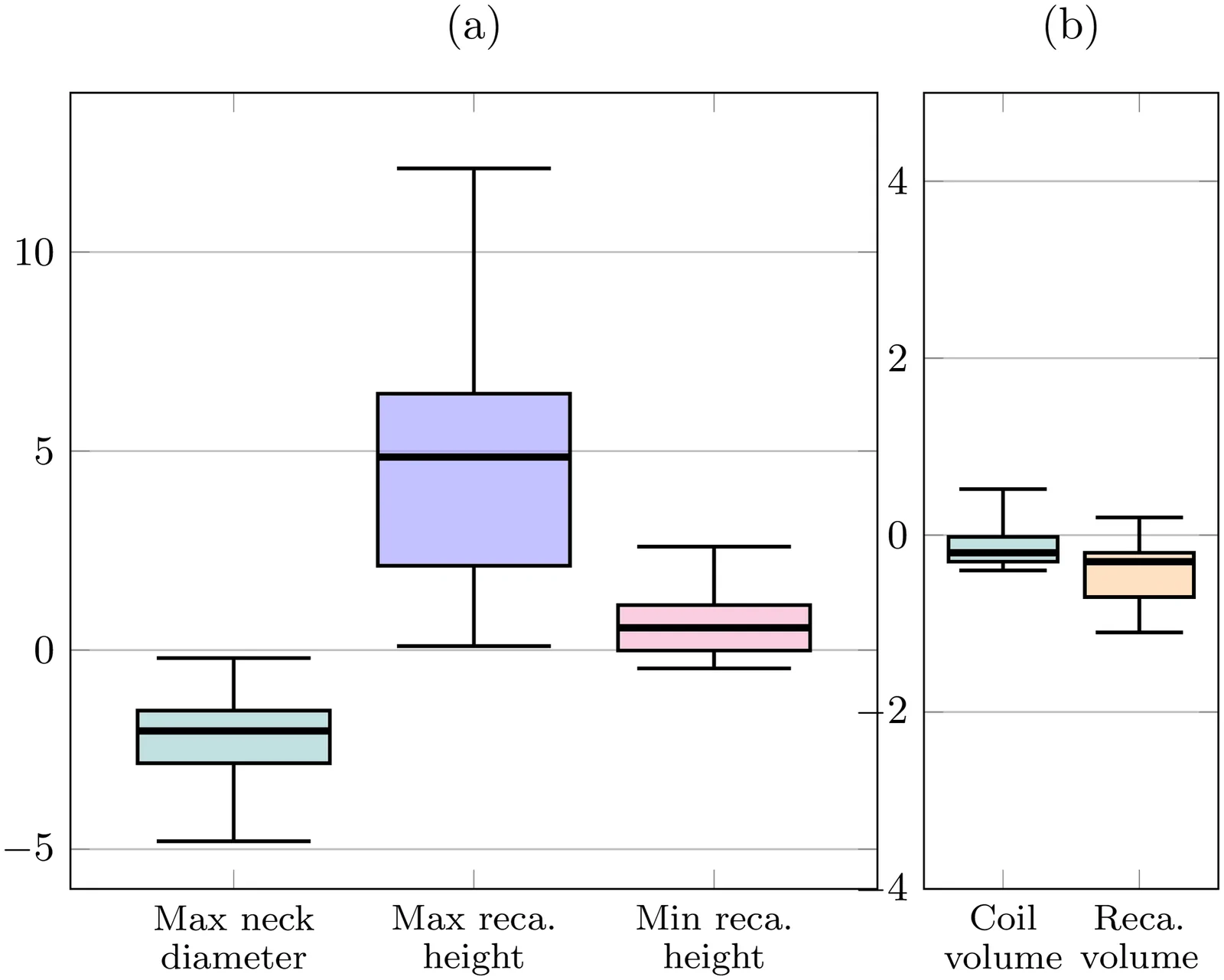
Comparison of manual and automatic measurements of: (a) neck/recanalization diameters and heights (mm) and (b) coil and recanalization volumes (mm^3^).

Fig 3 (a) presents the differences between manual and automatic measurements of the maximum neck diameter and the distance map (maximum and minimum heights between the recanalization and neck surfaces).

Fig 3 (b) illustrates the differences between manual and automatic volume measurements for both the coil volume and recanalization area volume. Here, greater variability was observed compared to the linear measurements, as indicated by a broader IQR and several outliers, particularly for the coil volume. Despite this variability, the median differences remained close to zero.

For the six cases with paired manual and automatic neck measurements, the mean absolute error in maximum neck diameter was 0.40 mm (median: 0.30 mm; range: 0–1.10 mm). The largest error corresponded to a complex aneurysm with a daughter sac, for which the automatic neck plane was incorrectly positioned.

## Discussion

### Segmentation

**Fig 4:**
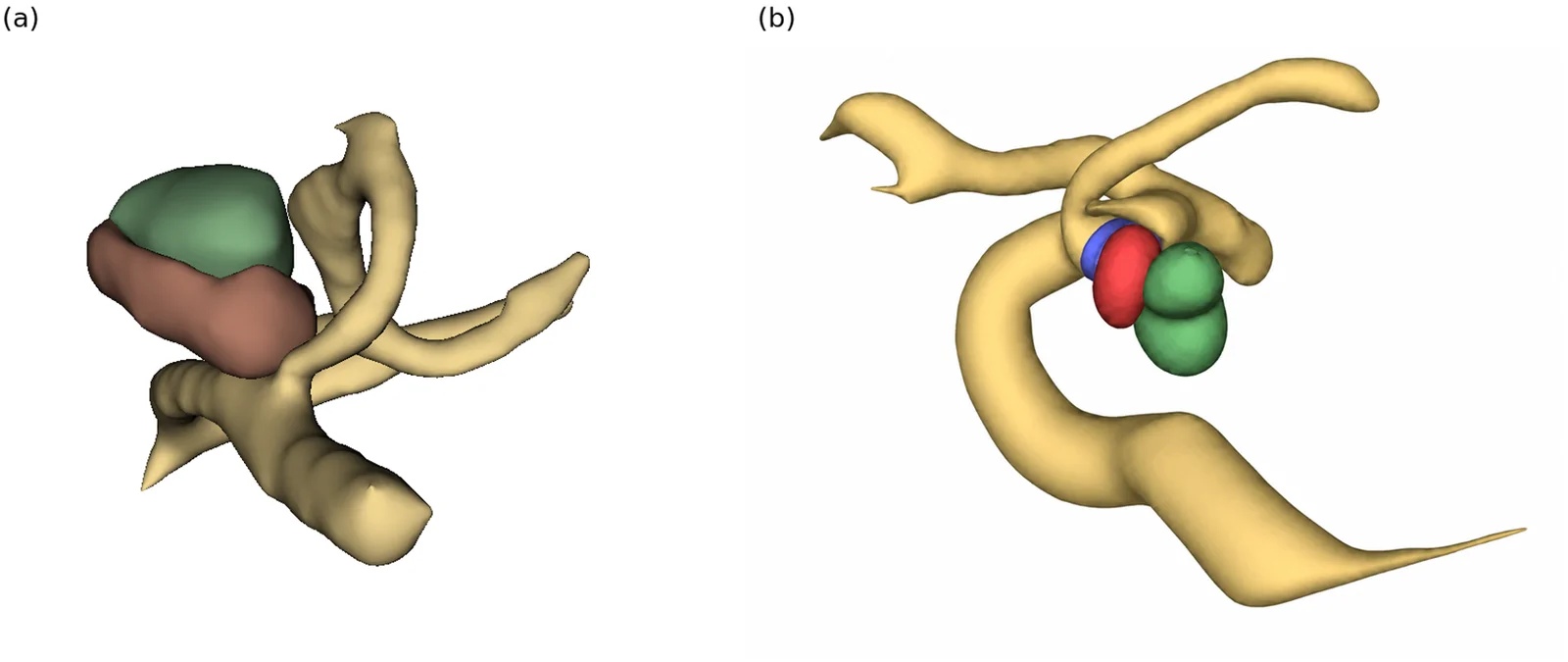
Representative 3D segmentation results. (a) Accurate segmentation of a coiled aneurysm. (b) Challenging case showing partial overlap between the parent vessel and the recanalized lumen. Parent vessel: yellow; recanalized lumen: red; coil mass: green; overlap: blue.

The average Dice and Jaccard scores obtained with nnU-Net are encouraging and confirm the potential of the approach. Lower scores were mainly observed for the recanalization area, which can be explained by its overlap with the vascular tree: both structures share similar signal characteristics, making them difficult to separate reliably. Fig 4b illustrates a representative case in which recanalization is partially merged with the vascular tree (in blue), while Fig 4a provides an example of accurate segmentation, highlighting the algorithm’s capacity when anatomical boundaries are well defined. Another factor contributing to reduced Dice values, particularly for the coil and vascular tree, is the presence of aneurysms with slightly elevated necks, which introduce geometric ambiguities and complicate segmentation accuracy. Increasing the congruence of the training database and including a wider variety of such challenging cases would likely improve the robustness of the model. The relatively limited cohort size reflects both the specificity of the clinical population and the absence of publicly available datasets addressing this particular setting. Eligible cases required a recanalized previously coiled aneurysm, suitable 3D-RA imaging, and expert three-dimensional annotations. Consequently, the present results should be regarded as a proof-of-concept evaluation, and larger multicentric cohorts will be required to assess generalizability.

Furthermore, missing branches in the vascular tree annotations likely confused the neural network. In several cases, small vessels were present in the scans but were not labeled, as they were considered clinically irrelevant. The network segmented these small vessels, which appear as false positives compared to incomplete ground truth, thus lowering the Dice score. The segmentation error is therefore slightly overestimated, mainly due to the complexity of the task and the fact that the manual annotations were not fully exhaustive. With more complete labeling, the Dice score would likely improve by a small margin.

Visual analysis of the 18 cases included in the downstream evaluation, based on neurosurgeon feedback, showed good-quality segmentations in 11 cases. Four cases exhibited minor imperfections, including slightly elevated necks, unusual aneurysm shapes, or small cropping errors, while three cases contained incomplete vascular reference annotations.

### Morphometry

**Fig 5:**
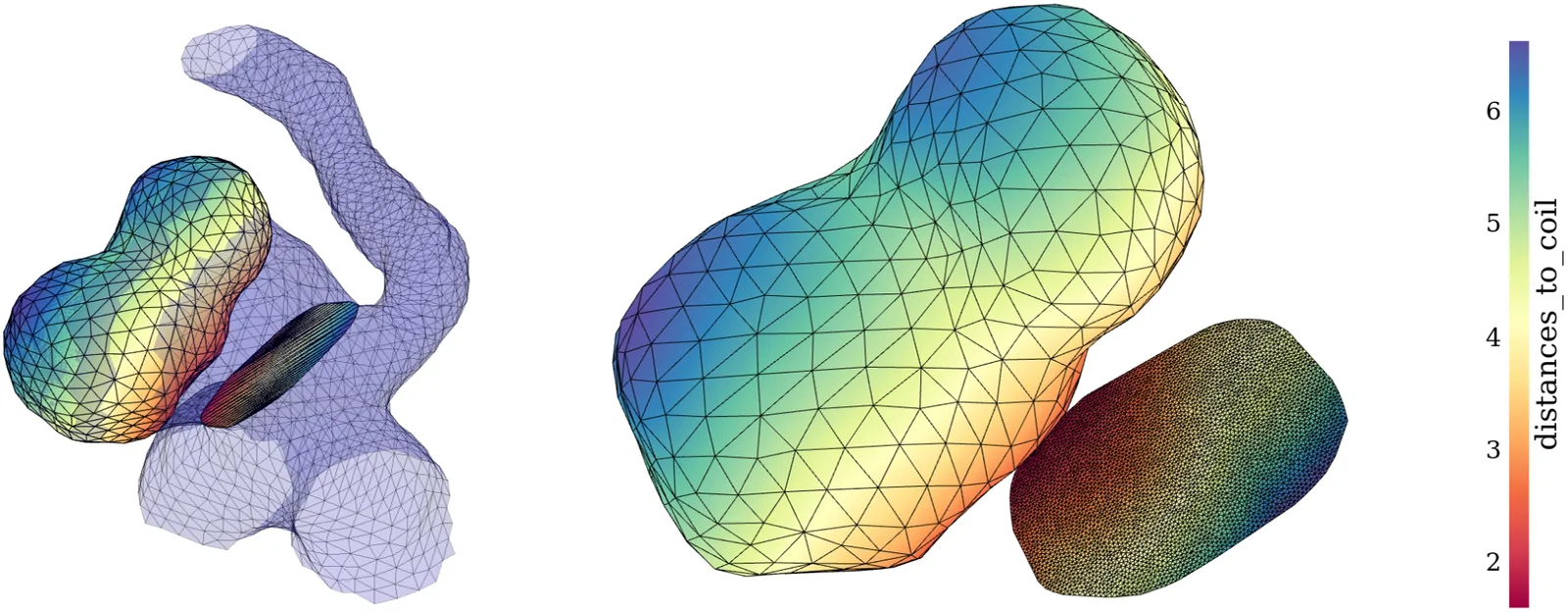
Patient with accurate neck placement. Map showing the distance from the neck of the aneurysm to the coil, measurements are in millimeters (mm).

**Fig 6:**
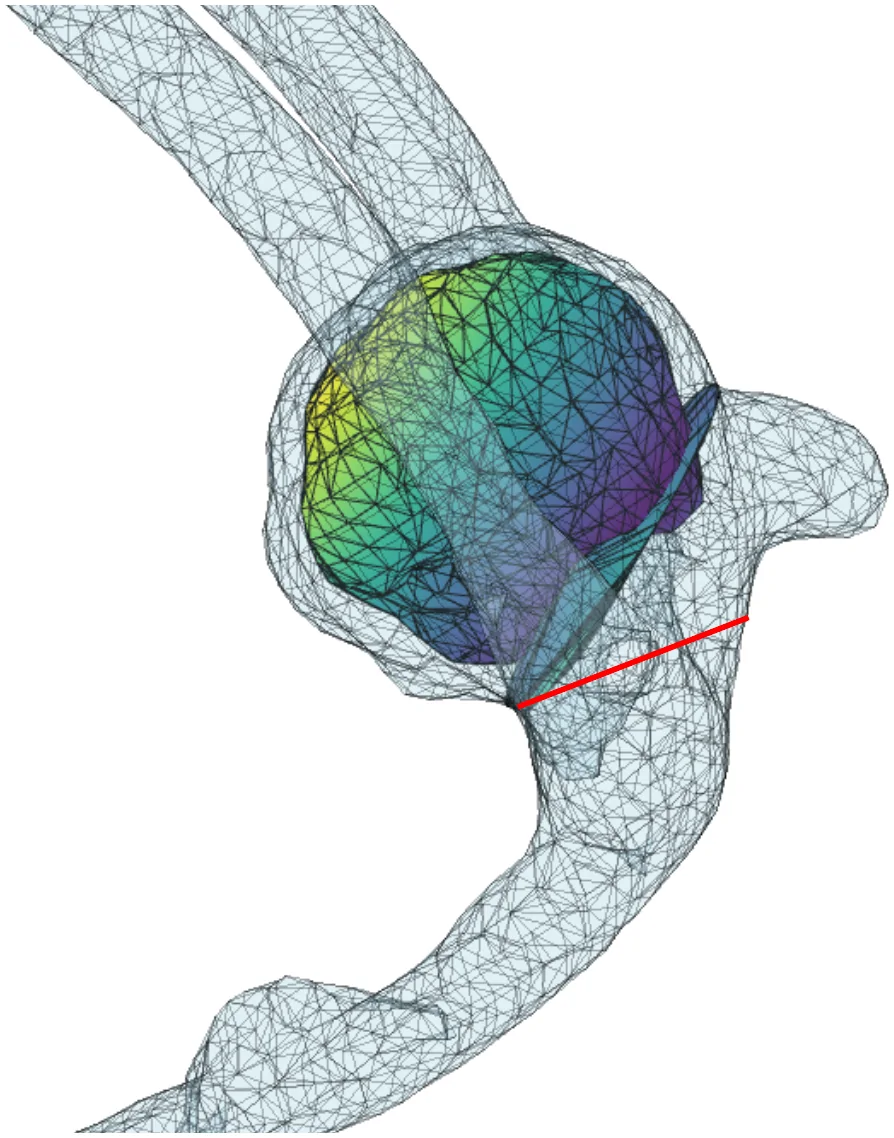
Patient with incorrect neck placement: the plane (blue disk across the coil) missed the daughter sac. The correct neck is highlighted in red.

The neck detection algorithm is based on a weighting process designed to minimize a cost function, which requires the adjustment of three parameters. Although in most cases the algorithm performs reliably without frequent parameter changes, situations where manual tuning is necessary can be time-consuming and may limit reproducibility. Fig 5 illustrates an accurately positioned neck plane, whereas Fig 6 shows an example of misestimation involving a recanalization with a daughter sac. This case highlights a limitation of the current algorithm for accurate neck placement. The correct definition of the neck plane is crucial, since it directly conditions all subsequent morphometric calculations, including diameters, heights, and volumes. An inaccurate placement can therefore propagate errors across the entire workflow. Jerman et al. [16] demonstrated the feasibility of accurate automatic neck-curve localization using direct geometric comparison with manual annotations. However, a direct comparison with their reported performance is not possible here, since equivalent geometric neck-curve metrics were not available for our cohort. The present dataset also introduces specific challenges, including coil artifacts and complex recanalization geometries, which make accurate definition of the cutting plane particularly difficult. Improving the robustness of this step is therefore a key priority, and efforts are currently underway in our laboratory to refine the algorithm and adapt it more specifically to these cases.

To assess the precision of the proposed method, the differences between manual expert annotations and automatic measurements were analyzed for both diameters/heights and volumetric parameters (Figure 3). Regarding the morphometric diameters and heights in Figure 3 (a), the algorithm showed a systematic tendency to slightly underestimate the maximum neck diameter compared with manual measurements. While this bias remained slight, it highlights the difficulty of precisely capturing the neck boundaries, which are often irregular. The minimum recanalization height, on the other hand, demonstrated very good concordance between manual and automatic estimates, suggesting that this parameter is robustly extracted by the model. The maximum recanalization height represented the most challenging measurement: the algorithm consistently overestimated this value and exhibited substantial variability across patients. This finding reflects the sensitivity of the segmentation to local geometric irregularities and the frequent difficulty of experts themselves in delineating the exact extent of the recanalized lumen. To overcome this limitation, a more accurate and robust definition of the neck plane would be beneficial. This aspect is currently under investigation in our ongoing work.

In terms of volumetric parameters from Figure 3 (b), the agreement was generally stronger. The coil volume was reproduced with high fidelity by the automatic approach, indicating that the model correctly identified and excluded the coil mass during segmentation. The volume of recanalization was generally slightly underestimated, but the magnitude of the difference remained limited in this proof-of-concept cohort. Together, these results suggest that the proposed method can provide consistent estimates for several morphometric parameters, while also highlighting that the estimation of neck-related features, particularly the maximum recanalization height, remains the most challenging aspect. These findings underscore the importance of including such metrics in validation studies, as they directly impact the evaluation of clipping feasibility and surgical planning.

### Limitations

The current model was trained on a relatively small dataset, consisting of 33 coiled and 30 non-coiled aneurysms. This limited size may reduce the model’s ability to generalize, especially to more complex or less common aneurysm presentations. Certain features, such as very small branches or highly irregular shapes, may be underrepresented in the current data. Increasing the number of coiled aneurysms in particular would help improve performance and robustness.

An additional limitation concerns the quantitative validation of the aneurysm neck detection step. Although the effect of neck-plane estimation was indirectly assessed through the comparison of manual and automatic maximum neck diameters in six cases, no direct geometric comparison between the manually defined and automatically estimated neck planes or contours was available. Future validation should therefore include dedicated geometric metrics, such as inter-curve distance, plane orientation error, or distance between manual and automatic neck centers, to more directly assess the accuracy and reproducibility of neck localization.

Finally, although the segmentation output is visually consistent in many cases, errors in aneurysm neck delineation can significantly impact morphometric parameters, particularly when necks are misidentified or poorly segmented, such as the case of the missing daughter sac observed in Fig. 6. This is especially relevant for clip planning, as neck delineation is a crucial step for the rest of the morphological assessment.

A further limitation concerns the reference annotations, which were produced by a single neurosurgeon. Consequently, inter-observer variability could not be assessed, and observer-dependent differences in the delineation of the recanalized lumen, aneurysm neck, or vascular structures may affect the reported performance. In addition, vascular-tree annotations were task-oriented and were not intended to constitute exhaustive anatomical segmentations, as some small collateral vessels or vessels considered irrelevant to clipping planning were not systematically labeled. This may lead to anatomically valid network predictions being counted as false positives. Future studies should include annotations from multiple independent experts to quantify inter-observer agreement and establish a more robust reference standard.

### Perspectives

To improve performance, we plan to construct a more balanced and diverse training dataset by including a wider range of aneurysm morphologies and sizes. This will improve generalization across varied clinical presentations.

We also aim to add post-processing steps such as coil extraction, using a threshold-based approach to remove the coil from segmentation to better distinguish between the recanalization area and adjacent cerebral vessels.

Further validation will include testing on larger and more diverse cohorts to assess robustness across a broader range of aneurysm morphologies. Particular attention will be given to the neck detection step, with direct geometric comparison between manual and automatic neck definitions using dedicated metrics such as inter-curve distance, plane orientation error, and neck-center displacement. These analyses will guide further optimization of the cost function and improve the reproducibility of morphometric parameter extraction for clipping assessment.

## Conclusion

This study presents a proof-of-concept automated workflow for the segmentation and morphometric analysis of recanalized coiled intracranial aneurysms from 3D-RA images. By combining nnU-Net-based segmentation with the extraction of morphological parameters relevant to microsurgical clipping assessment, the proposed framework provides a promising basis for more objective and reproducible preoperative evaluation. The initial results demonstrate the potential of the approach to support surgical planning, while larger multicentric validation and further improvement of aneurysm neck detection will be required before clinical implementation.

## References

[1] Brain Aneurysm Foundation. Statistics and Facts; 2023. Available from: https://www.bafound.org/statistics-and-facts/

[2] Andrew M, Jersey DMF. Cerebral Aneurysm; 2023. Available from: https://www.ncbi.nlm.nih.gov/books/NBK507902/

[3] dos Santos MP, Sabri A, Dowlatshahi D, Bakkai AM, Elallegy A, Lesiuk H, et al. Survival analysis of risk factors for major recurrence of intracranial aneurysms after coiling. Can J Neurol Sci. 2015;42(1):40–7. doi: 10.1017/cjn.2014.126 25635401

[4] Brinjikji W, White PM, Nahser H, Wardlaw J, Sellar R, Gholkar A, et al. HydroCoils Are Associated with Lower Angiographic Recurrence Rates Than Are Bare Platinum Coils in Treatment of “Difficult-to-Treat” Aneurysms: A Post Hoc Subgroup Analysis of the HELPS Trial. AJNR Am J Neuroradiol. 2015;36(9):1689–94. doi: 10.3174/ajnr.A4349 26228887 PMC7968763

[5] Son Y-J, Kwon O-K, Hwang G, Park NM, Oh CW, Bang JS. Major recanalization occurs more often in young patients after unruptured aneurysm coil embolization. Acta Neurochir (Wien). 2016;158(3):551–6. doi: 10.1007/s00701-015-2668-1 26743913

[6] Stapleton CJ, Torok CM, Rabinov JD, Walcott BP, Mascitelli JR, Leslie-Mazwi TM, et al. Validation of the Modified Raymond-Roy classification for intracranial aneurysms treated with coil embolization. J Neurointerv Surg. 2016;8(9):927–33. doi: 10.1136/neurintsurg-2015-012035 26438554

[7] Briganti F, Tortora M, Loiudice G, Tarantino M, Guida A, Buono G, et al. Utility of virtual stenting in treatment of cerebral aneurysms by flow diverter devices. Radiol Med. 2023;128(4):480–91. doi: 10.1007/s11547-023-01620-x 37027092

[8] Mensah E, Pringle C, Roberts G, Gurusinghe N, Golash A, Alalade AF. Deep Learning in the Management of Intracranial Aneurysms and Cerebrovascular Diseases: A Review of the Current Literature. World Neurosurg. 2022;161:39–45. doi: 10.1016/j.wneu.2022.02.006 35134582

[9] Di Noto T, Marie G, Tourbier S, Alemán-Gómez Y, Esteban O, Saliou G, et al. Towards Automated Brain Aneurysm Detection in TOF-MRA: Open Data, Weak Labels, and Anatomical Knowledge. Neuroinformatics. 2023;21(1):21–34. doi: 10.1007/s12021-022-09597-0 35982364 PMC9931814

[10] Liu X, Mao J, Sun N, Yu X, Chai L, Tian Y, et al. Deep Learning for Detection of Intracranial Aneurysms from Computed Tomography Angiography Images. J Digit Imaging. 2023;36(1):114–23. doi: 10.1007/s10278-022-00698-5 36085330 PMC9984635

[11] Bo Z-H, Qiao H, Tian C, Guo Y, Li W, Liang T, et al. Toward human intervention-free clinical diagnosis of intracranial aneurysm via deep neural network. Patterns (N Y). 2021;2(2):100197. doi: 10.1016/j.patter.2020.100197 33659913 PMC7892358

[12] Berg P, Voß S, Saalfeld S, Janiga G, Bergersen AW, Valen-Sendstad K, et al. Multiple Aneurysms AnaTomy CHallenge 2018 (MATCH): Phase I: Segmentation. Cardiovasc Eng Technol. 2018;9(4):565–81. doi: 10.1007/s13239-018-00376-0 30191538

[13] Timmins KM, van der Schaaf IC, Bennink E, Ruigrok YM, An X, Baumgartner M, et al. Comparing methods of detecting and segmenting unruptured intracranial aneurysms on TOF-MRAS: The ADAM challenge. Neuroimage. 2021;238:118216. doi: 10.1016/j.neuroimage.2021.118216 34052465

[14] Ivantsits M, Goubergrits L, Kuhnigk J-M, Huellebrand M, Bruening J, Kossen T, et al. Detection and analysis of cerebral aneurysms based on X-ray rotational angiography - the CADA 2020 challenge. Med Image Anal. 2022;77:102333. doi: 10.1016/j.media.2021.102333 34998111

[15] Nishi H, Cancelliere NM, Rustici A, Charbonnier G, Chan V, Spears J, et al. Deep learning-based cerebral aneurysm segmentation and morphological analysis with three-dimensional rotational angiography. J Neurointerv Surg. 2024;16(2):197–203. doi: 10.1136/jnis-2023-020192 37192786

[16] Jerman T, Chien A, Pernus F, Likar B, Spiclin Z. Automated Cutting Plane Positioning for Intracranial Aneurysm Quantification. IEEE Trans Biomed Eng. 2020;67(2):577–87. doi: 10.1109/TBME.2019.2918921 31144619

[17] Saalfeld S, Berg P, Niemann A, Luz M, Preim B, Beuing O. Semiautomatic neck curve reconstruction for intracranial aneurysm rupture risk assessment based on morphological parameters. Int J Comput Assist Radiol Surg. 2018;13(11):1781–93. doi: 10.1007/s11548-018-1848-x 30159832

[18] Ma J, Nie Z. Exploring large context for cerebral aneurysm segmentation. In: Cerebral Aneurysm Detection and Analysis: First Challenge, CADA 2020, Held in Conjunction with MICCAI 2020, Lima, Peru, October 8, 2020, Proceedings 1. Springer; 2021. p. 68–72.

[19] Müller D, Soto-Rey I, Kramer F. Towards a guideline for evaluation metrics in medical image segmentation. BMC Res Notes. 2022;15(1):210. doi: 10.1186/s13104-022-06096-y 35725483 PMC9208116

