# Supplementary material for: Segmentation and morphological analysis of recanalized coiled aneurysms: Towards an automatic tool for clipping planning in microsurgery
Source: PLoS One. 2026 Sep 23;21(9):e0358945. doi: 10.1371/journal.pone.0358945 (PMC13600480; doi:10.1371/journal.pone.0358945)
Supplement: Supplementary file 1 [file pone.0358945.s001.docx]

Response to Reviewers

| **Manuscript ID** | PONE-D-26-03376 |
| --- | --- |
| **Journal** | PLOS ONE |
| **Title** | Segmentation and Morphological Analysis of Recanalized Coiled Aneurysms: Towards an Automatic Tool for Clipping Planning in Microsurgery |
| **Decision** | Revision required |

# General response

We thank the Academic Editor and Reviewer #1 for their careful evaluation of our manuscript and for the constructive comments. We have revised the manuscript accordingly and provide a point-by-point response below.

In addition to addressing the reviewer’s comments, we have also implemented the additional editorial requirements. The manuscript has been reformatted using the appropriate PLOS ONE template and the figures and associated files have been revised to comply with the journal’s formatting requirements. We have also revised the Data Availability Statement to provide a non-author institutional contact point for data access requests. The revised statement is:

“The data underlying the results presented in this study are not publicly available due to ethical and legal restrictions related to patient confidentiality. The data are held by Brest University Hospital (CHU de Brest, France) within the PREDICTNRI study (ClinicalTrials.gov identifier NCT04504695). Qualified researchers may submit data access requests to the institutional research-on-data unit of Brest University Hospital (Direction de la Recherche Clinique et de l’Innovation, cellule.rniph@chu-brest.fr). Access is subject to applicable institutional, ethical, and legal approvals.”

The ethics statement has been retained exclusively within the Methods section, as requested.

# Reviewer #1 - Major comments

## Major comment 1 - Novelty claim and literature search

**Reviewer comment:** *The authors claim this is the first fully automated method for analyzing recanalized coiled aneurysms. While the application is indeed novel, this claim should be better substantiated with a database article research and statement of the key words used.*

**Response**

We thank the reviewer for this important comment. We agree that the novelty claim should be supported by a more explicit and reproducible literature search and that the wording should be more precise.

To address this point, we performed a targeted PubMed literature search on August 12, 2026, using combinations of the following keywords: *“intracranial aneurysm”, “cerebral aneurysm”, “coiled/coiling”, “embolized/embolised”, “recanalization/recanalisation”, “recurrence”, “segmentation”, “morphometry”, “morphological analysis”, “neck detection”, “automatic/automated”, “deep learning”, “machine learning”, “3D rotational angiography/3D-RA”, “digital subtraction angiography/DSA”, “clipping”, “microsurgery/microsurgical”,* and *“surgical planning”*.

The initial search combining aneurysm, coiling/embolization, and recanalization/recurrence terms returned 2,247 records. Adding segmentation, morphometric analysis, neck detection, or surgical-planning terms reduced this to 645 records. A more restrictive search including automated or AI-based analysis returned only 2 records, while the search specifically combining automation with clipping/surgical planning returned 1 record, which was unrelated to automated image analysis. Finally, a modality-specific search combining coiled/recanalized aneurysms, segmentation or morphometric analysis, and 3D rotational/digital subtraction angiography returned 41 records.

Screening of these targeted results identified studies addressing aneurysm volume measurement, recurrence assessment, morphometric predictors of recurrence, and computational fluid-dynamic prediction after coiling. However, none described an equivalent framework combining segmentation and morphometric analysis of recanalized coiled aneurysms from 3D rotational angiography specifically for microsurgical clipping planning.

We therefore revised the manuscript to explicitly report the literature-search strategy and to narrow the novelty statement to this specific clinical and methodological setting. We also removed the term *“fully”* from the claim to avoid overstating the degree of automation of the current workflow.

The revised Introduction now includes the following text:

**Revised manuscript text**

**Introduction:** *“A targeted PubMed search using combinations of terms related to intracranial aneurysms, coiling, recanalization, segmentation, morphometry, automation, 3D rotational angiography, clipping, and surgical planning identified no equivalent framework specifically addressing automated segmentation and morphometric analysis of recanalized coiled aneurysms for microsurgical clipping planning. The main contributions of this work are: (i) an automated nnU-Net-based segmentation of the vascular tree, coil mass, and recanalized lumen from 3D-RA images; and (ii) a morphometric analysis of the recanalized aneurysm, including neck characterization, coil-to-neck distance, and recanalization volume, designed to support preoperative assessment for microsurgical clipping.”*

## Major comment 2 - Small dataset and uncertainty estimates

**Reviewer comment:** *The dataset is relatively small: Provide justification (e.g., the rarity of data), add confidence intervals or variability measures.*

**Response**

We thank the reviewer for highlighting this limitation. We agree that the cohort size is relatively small. However, the population investigated in this study corresponds to a highly specific clinical scenario, and suitable data are relatively rare. Eligible cases required a previously coiled intracranial aneurysm presenting recanalization, together with complete and exploitable 3D rotational angiography available for retrospective analysis. In addition, each case required expert three-dimensional annotation of the vascular structures, coil mass, and recanalized compartment. The combination of these criteria substantially restricts the number of eligible cases and makes the constitution of such a dataset particularly demanding.

We have therefore revised the manuscript to better emphasize that the present study constitutes a proof-of-concept evaluation of the proposed framework rather than a definitive clinical validation. The limited sample size may particularly affect the representation of uncommon or complex aneurysm morphologies, and validation on larger, ideally multicentric cohorts will be necessary to assess the generalizability of the proposed approach.

Following the reviewer’s suggestion, we also expanded the quantitative reporting by adding 95% confidence intervals for all Dice and Jaccard scores obtained on the independent segmentation test set of 16 cases. Mean Dice scores were 0.878 (95% CI: 0.783–0.943) for the coil, 0.876 (95% CI: 0.779–0.923) for the vascular tree, and 0.783 (95% CI: 0.693–0.822) for the recanalization area. Corresponding mean Jaccard indices were 0.788 (95% CI: 0.769–0.876), 0.790 (95% CI: 0.774–0.869), and 0.659 (95% CI: 0.633–0.801), respectively. These confidence intervals have been added to Table 1. Variability in the subsequent morphometric evaluation is additionally illustrated through the distribution of differences between manual and automatic measurements.

**Revised manuscript text**

**Database:** *“To our knowledge, no publicly available dataset provides 3D-RA images of recanalized coiled aneurysms together with expert three-dimensional annotations of the vascular tree, coil mass, and recanalized compartment. The dataset therefore had to be assembled retrospectively from this highly specific clinical population, substantially limiting the number of eligible cases.”*

**Discussion:** *“Consequently, the present results should be regarded as a proof-of-concept evaluation, and larger multicentric cohorts will be required to assess generalizability.”*

**Table 1:** *“Segmentation performance on the independent test set (n = 16). Values are reported as mean with 95% confidence intervals (CI).”*

## Major comment 3 - Ground truth and inter-observer variability

**Reviewer comment:** *Ground Truth Limitations: Manual annotations (performed by a single neurosurgeon with no inter-observer variability assessment), please discuss.*

**Response**

We thank the reviewer for raising this important point. All manual reference annotations used in this study were performed by a single qualified neurosurgeon with expertise in cerebrovascular surgery. As no second independent annotation was available, inter-observer variability could not be assessed. We agree that this represents a limitation, since the delineation of the aneurysm neck, recanalized lumen, and vascular structures may be subject to observer-dependent variability.

We have therefore clarified the annotation procedure in the Methods and explicitly added the absence of inter-observer assessment to the Limitations section. We also clarified that the vascular-tree annotations were task-oriented rather than intended to provide an exhaustive anatomical segmentation: vascular structures considered relevant to aneurysm assessment and clipping planning were prioritized, whereas some small collateral vessels or vessels crossing the region of interest were not systematically annotated. Consequently, some vessels correctly identified by the network may appear as false positives when compared with the reference annotations.

Future validation should therefore include annotations from multiple independent experts, allowing inter-observer agreement to be quantified and providing a more robust reference standard for both segmentation and morphometric evaluation.

**Revised manuscript text**

**Limitations:** *“A further limitation concerns the reference annotations, which were produced by a single neurosurgeon. Consequently, inter-observer variability could not be assessed, and observer-dependent differences in the delineation of the recanalized lumen, aneurysm neck, or vascular structures may affect the reported performance. Future studies should include annotations from multiple independent experts to quantify inter-observer agreement and establish a more robust reference standard.”*

## Major comment 4 - Quantitative validation of neck detection

**Reviewer comment:** *The neck detection step works well in many cases, but it seems less robust for more complex aneurysm shapes and sometimes requires parameter adjustment. Since all morphological measurements depend on this step, improving its reliability could further strengthen the overall method. It would be helpful to include a quantitative evaluation of neck detection accuracy.*

**Response**

We thank the reviewer for this important comment. We agree that the neck detection step is a critical component of the workflow, since errors in neck-plane positioning can propagate to subsequent neck-related morphometric measurements.

To provide a quantitative assessment of this step, we added a comparison between manual and automatic maximum neck diameter measurements for six cases for which paired measurements were available. The mean absolute error was 0.40 mm, with a median absolute error of 0.30 mm and a range of 0–1.10 mm. The largest discrepancy (1.10 mm) corresponded to a complex aneurysm with a daughter sac, for which the automatically detected neck plane was incorrectly positioned.

We acknowledge that this evaluation remains limited in size and assesses neck detection indirectly through the resulting neck diameter rather than through a direct geometric comparison of the manual and automatic neck contours. This limitation has now been explicitly stated in the manuscript. A more comprehensive validation using direct geometric metrics, such as the average inter-curve distance between manual and automatic neck contours, will be required in future studies.

**Revised manuscript text**

**Results:** *“For the six cases with paired manual and automatic neck measurements, the mean absolute error in maximum neck diameter was 0.40 mm (median: 0.30 mm; range: 0–1.10 mm). The largest error corresponded to a complex aneurysm with a daughter sac, for which the automatic neck plane was incorrectly positioned.”*

**Limitations:** *“Although the effect of neck-plane estimation was indirectly assessed through the comparison of manual and automatic maximum neck diameters in six cases, no direct geometric comparison between the manually defined and automatically estimated neck planes or contours was available. Future validation should therefore include dedicated geometric metrics to more directly assess the accuracy and reproducibility of neck localization.”*

# Reviewer #1 - Minor comments

## Minor comment 1 - Language and terminology

**Reviewer comment:** *Several grammatical issues (e.g., 'recanalysation' vs 'recanalization').*

**Response**

We thank the reviewer. We have revised the manuscript for language and consistency and standardized the terminology throughout. In particular, 'recanalization' is now used consistently, and grammatical and typographical errors have been corrected.

## Minor comment 2 - Figure quality and labeling

**Reviewer comment:** *Improve resolution and labelling of the Figures.*

**Response**

We thank the reviewer for this suggestion. We have revised the figures throughout the manuscript to improve their resolution, panel labeling, font size, and overall visual consistency. In particular, Figure 4 has been substantially redesigned, as the previous version combined heterogeneous 2D and 3D renderings with different backgrounds and relatively low-resolution screenshots. The revised figure now uses a more consistent presentation, clearer labels, harmonized color coding, and higher-quality 3D visualizations to better illustrate both an accurate segmentation and a challenging case.

## Minor comment 3 - Related work on treatment planning

**Reviewer comment:** *The manuscript could benefit from citing related work on computational tools for treatment planning of cerebral aneurysms. For example, the study by Briganti et al. (2023) on virtual stenting highlights the value of simulation-based approaches in supporting clinical decision-making.*

**Response**

We thank the reviewer for this suggestion. We have added the study by Briganti et al. (2023) to the Introduction to broaden the discussion of computational tools for cerebral aneurysm treatment planning. The cited work on virtual stenting illustrates the potential of simulation-based approaches to support clinical decision-making and provides complementary context for the present segmentation and morphometric planning framework.

**Revised manuscript text**

**Introduction:** *“More broadly, computational tools have also been investigated to support cerebral aneurysm treatment planning. For example, Briganti et al. (2023) explored virtual stenting as a simulation-based approach to assist endovascular treatment planning and clinical decision-making.”*
