# Supplementary material for: Segmentation and morphological analysis of recanalized coiled aneurysms: Towards an automatic tool for clipping planning in microsurgery
Source: PLoS One. 2026 Sep 23;21(9):e0358945. doi: 10.1371/journal.pone.0358945 (PMC13600480; doi:10.1371/journal.pone.0358945)
Supplement: Peer review history

# Peer review: Decision Letter 0

**DOI:** 10.1371/journal.pone.0358945.r001

**Authors:** Mario Tortora

19 Apr 2026

Dear Dr. Matthieu,

Thank you for submitting your manuscript to PLOS ONE. After careful consideration, we feel that it has merit but does not fully meet PLOS ONE’s publication criteria as it currently stands. Therefore, we invite you to submit a revised version of the manuscript that addresses the points raised during the review process.

Please submit your revised manuscript by Jun 03 2026 11:59PM. If you will need more time than this to complete your revisions, please reply to this message or contact the journal office at plosone@plos.org. When you're ready to submit your revision, log on to https://www.editorialmanager.com/pone/ and select the 'Submissions Needing Revision' folder to locate your manuscript file.

- A letter that responds to each point raised by the academic editor and reviewer(s). You should upload this letter as a separate file labeled 'Response to Reviewers'.
- A marked-up copy of your manuscript that highlights changes made to the original version. You should upload this as a separate file labeled 'Revised Manuscript with Track Changes'.
- An unmarked version of your revised paper without tracked changes. You should upload this as a separate file labeled 'Manuscript'.

If you would like to make changes to your financial disclosure, please include your updated statement in your cover letter. Guidelines for resubmitting your figure files are available below the reviewer comments at the end of this letter.

If applicable, we recommend that you deposit your laboratory protocols in protocols.io to enhance the reproducibility of your results. Protocols.io assigns your protocol its own identifier (DOI) so that it can be cited independently in the future. For instructions see: https://journals.plos.org/plosone/s/submission-guidelines#loc-laboratory-protocols. Additionally, PLOS ONE offers an option for publishing peer-reviewed Lab Protocol articles, which describe protocols hosted on protocols.io. Read more information on sharing protocols at https://plos.org/protocols?utm_medium=editorial-email&utm_source=authorletters&utm_campaign=protocols.

As the corresponding author, your ORCID iD is verified in the submission system and will appear in the published article. PLOS supports the use of ORCID, and we encourage all coauthors to register for an ORCID iD and use it as well. Please encourage your coauthors to verify their ORCID iD within the submission system before final acceptance, as unverified ORCID iDs will not appear in the published article. *Only*  the individual author can complete the verification step; PLOS staff *cannot*  verify ORCID iDs on behalf of authors.

We look forward to receiving your revised manuscript.

Kind regards,

Mario Tortora, Pd.D.s, M.D.

Academic Editor

PLOS One

Journal Requirements:

When submitting your revision, we need you to address these additional requirements.

1.Please ensure that your manuscript meets PLOS ONE's style requirements, including those for file naming. The PLOS ONE style templates can be found at

https://journals.plos.org/plosone/s/file?id=wjVg/PLOSOne_formatting_sample_main_body.pdf and

https://journals.plos.org/plosone/s/file?id=ba62/PLOSOne_formatting_sample_title_authors_affiliations.pdf

2. Please note that PLOS One has specific guidelines on code sharing for submissions in which author-generated code underpins the findings in the manuscript. In these cases, we expect all author-generated code to be made available without restrictions upon publication of the work. Please review our guidelines at https://journals.plos.org/plosone/s/materials-and-software-sharing#loc-sharing-code and ensure that your code is shared in a way that follows best practice and facilitates reproducibility and reuse.

3. In the online submission form you indicate that your data is not available for proprietary reasons and have provided a contact point for accessing this data. Please note that your current contact point is a co-author on this manuscript. According to our Data Policy, the contact point must not be an author on the manuscript and must be an institutional contact, ideally not an individual. Please revise your data statement to a non-author institutional point of contact, such as a data access or ethics committee, and send this to us via return email. Please also include contact information for the third party organization, and please include the full citation of where the data can be found.

4. Your ethics statement should only appear in the Methods section of your manuscript. If your ethics statement is written in any section besides the Methods, please delete it from any other section.

5.If the reviewer comments include a recommendation to cite specific previously published works, please review and evaluate these publications to determine whether they are relevant and should be cited. There is no requirement to cite these works unless the editor has indicated otherwise.

Additional Editor Comments:

Dear Authors,

Following the evaluation of your manuscript, I would like to share with you the comments received from the reviewer.

Overall, the work shows potential and addresses relevant aspects of the topic; however, several points have been raised that require your careful attention before we can proceed further in the editorial process.

I therefore encourage you to revise the manuscript thoroughly, taking into account all the reviewer’s comments.

A careful and comprehensive revision in line with these comments will facilitate a more efficient and timely evaluation of your work.

Please do not hesitate to contact me should you need any clarification.

I look forward to receiving your revised manuscript.

Kind regards,

Mario Tortora MD PhD

[Note: HTML markup is below. Please do not edit.]

Reviewers' comments:

Reviewer's Responses to Questions

**Comments to the Author**

1. Is the manuscript technically sound, and do the data support the conclusions?

Reviewer #1: Yes

**********

2. Has the statistical analysis been performed appropriately and rigorously?

Reviewer #1: Yes

**********

3. Have the authors made all data underlying the findings in their manuscript fully available?

The PLOS Data policy

Reviewer #1: No

**********

4. Is the manuscript presented in an intelligible fashion and written in standard English?

Reviewer #1: Yes

**********

Reviewer #1: Dear authors, I read with interest this manuscript which presents an automated framework for segmentation and morphological analysis of recanalized coiled intracranial aneurysms using nnU-Net applied to 3D rotational angiography (3D-RA). The study aims to support microsurgical clipping planning by extracting clinically relevant morphometric parameters such as recanalization volume, neck diameter, and coil–neck distance.

Major comments:

1) The authors claim this is the first fully automated method for analyzing recanalized coiled aneurysms. While the application is indeed novel, this claim should be better substantiated with a database article research and statement of the key words used

2) The dataset is relatively small: Provide justification (e.g.,thee rarity of data), add confidence intervals or variability measures..

3) Ground Truth Limitations: Manual annotations (performed by a single neurosurgeon with no inter-observer variability assessment, please discuss

4) The neck detection step works well in many cases, but it seems less robust for more complex aneurysm shapesand sometimes requires parameter adjustment. Since all morphological measurements depend on this step, improving its reliability could further strengthen the overall method. would be helpful to include a quantitative evaluation of neck detection accuracy

Minor Comments:

1) Several grammatical issues (e.g., “recanalysation” vs “recanalization”)

2) Improve resolution and labelling of the Figures

3) The manuscript could benefit from citing related work on computational tools for treatment planning of cerebral aneurysms. For example, the study by Briganti et al. (2023) (https://doi.org/10.1007/s11547-023-01620-x) on virtual stenting highlights the value of simulation-based approaches in supporting clinical decision-making.

**********

what does this mean?). If published, this will include your full peer review and any attached files.

If you choose “no”, your identity will remain anonymous but your review may still be made public.

**Do you want your identity to be public for this peer review?**  For information about this choice, including consent withdrawal, please see our Privacy Policy

Reviewer #1: No

**********

[NOTE: If reviewer comments were submitted as an attachment file, they will be attached to this email and accessible via the submission site. Please log into your account, locate the manuscript record, and check for the action link "View Attachments". If this link does not appear, there are no attachment files.]

To ensure your figures meet our technical requirements, please review our figure guidelines: https://journals.plos.org/plosone/s/figures

You may also use PLOS’s free figure tool, NAAS, to help you prepare publication quality figures: https://journals.plos.org/plosone/s/figures#loc-tools-for-figure-preparation.

NAAS will assess whether your figures meet our technical requirements by comparing each figure against our figure specifications.

# Author comment: Author response to Decision Letter 1

**DOI:** 10.1371/journal.pone.0358945.r002

7 Sep 2026

We thank the Academic Editor and Reviewer #1 for their careful evaluation of our manuscript and for their constructive comments. We have revised the manuscript accordingly, and a detailed point-by-point response to all reviewer comments is provided in the accompanying Response to Reviewers document.

In particular, we have: (i) substantiated and narrowed the novelty statement through a targeted PubMed literature search; (ii) clarified the limitations associated with the cohort size and added 95% confidence intervals for the segmentation metrics; (iii) explicitly described the use of a single expert reference annotation and discussed the absence of inter-observer variability assessment; (iv) added a quantitative evaluation of neck-related measurements and expanded the discussion of the limitations of neck detection; (v) improved figure quality and labeling; and (vi) added the relevant work by Briganti et al. (2023).

We have also addressed the additional editorial requirements. The manuscript has been reformatted according to the PLOS ONE template and figure/file naming conventions. The ethics statement appears only in the Methods section. To facilitate editorial processing, we have provided both manuscript versions (with and without embedded figures), as well as the individual figure files in the requested format.

The Data Availability Statement has also been revised to provide a non-author institutional contact point:

“The data underlying the results presented in this study are not publicly available due to ethical and legal restrictions related to patient confidentiality. The data are held by Brest University Hospital (CHU de Brest, France) within the PREDICTNRI study (ClinicalTrials.gov identifier NCT04504695). Qualified researchers may submit data access requests to the institutional research-on-data unit of Brest University Hospital (Direction de la Recherche Clinique et de l’Innovation, cellule.rniph@chu-brest.fr). Access is subject to applicable institutional, ethical, and legal approvals.”

We thank the Editor and Reviewer again for their comments, which have helped us improve the clarity and rigor of the manuscript.

Attachment Submitted filename: Response_to_Reviewers_PONE-D-26-03376_FINAL.docx

# Editor report: Decision Letter 1

**DOI:** 10.1371/journal.pone.0358945.r003

**Authors:** Mario Tortora

8 Sep 2026

Segmentation and Morphological Analysis of Recanalized Coiled Aneurysms: Towards an Automatic Tool for Clipping Planning in Microsurgery

PONE-D-26-03376R1

Dear Dr. Coupet,

We’re pleased to inform you that your manuscript has been judged scientifically suitable for publication and will be formally accepted for publication once it meets all outstanding technical requirements.

Within one week, you’ll receive an e-mail detailing the required amendments. When these have been addressed, you’ll receive a formal acceptance letter and your manuscript will be scheduled for publication.

An invoice will be generated when your article is formally accepted. Please note, if your institution has a publishing partnership with PLOS and your article meets the relevant criteria, all or part of your publication costs will be covered. Please make sure your user information is up-to-date by logging into Editorial Manager at Editorial Manager® and clicking the ‘Update My Information' link at the top of the page. For questions related to billing, please contact billing support.

If your institution or institutions have a press office, please notify them about your upcoming paper to help maximize its impact. If they’ll be preparing press materials, please inform our press team as soon as possible -- no later than 48 hours after receiving the formal acceptance. Your manuscript will remain under strict press embargo until 2 pm Eastern Time on the date of publication. For more information, please contact onepress@plos.org.

Kind regards,

Mario Tortora, Ph.D, M.D.

Academic Editor

PLOS One

Additional Editor Comments (optional):

Reviewers' comments:

# Editor report: Acceptance letter

**DOI:** 10.1371/journal.pone.0358945.r004

**Authors:** Mario Tortora

PONE-D-26-03376R1

PLOS One

Dear Dr. Coupet,

I'm pleased to inform you that your manuscript has been deemed suitable for publication in PLOS One. Congratulations! Your manuscript is now being handed over to our production team.

At this stage, our production department will prepare your paper for publication. This includes ensuring the following:

* All references, tables, and figures are properly cited

* All relevant supporting information is included in the manuscript submission,

* There are no issues that prevent the paper from being properly typeset

You will receive further instructions from the production team, including instructions on how to review your proof when it is ready. Please keep in mind that we are working through a large volume of accepted articles, so please give us a few days to review your paper and let you know the next and final steps.

Lastly, if your institution or institutions have a press office, please let them know about your upcoming paper now to help maximize its impact. If they'll be preparing press materials, please inform our press team within the next 48 hours. Your manuscript will remain under strict press embargo until 2 pm Eastern Time on the date of publication. For more information, please contact onepress@plos.org.

You will receive an invoice from PLOS for your publication fee after your manuscript has reached the completed accept phase. If you receive an email requesting payment before acceptance or for any other service, this may be a phishing scheme. Learn how to identify phishing emails and protect your accounts at https://explore.plos.org/phishing.

If we can help with anything else, please email us at customercare@plos.org.

Thank you for submitting your work to PLOS One and supporting open access.

Kind regards,

PLOS One Editorial Office Staff

on behalf of

Dr. Mario Tortora

Academic Editor

PLOS One
